# Quantitative assessment of left ventricular longitudinal function and myocardial deformation in Duchenne muscular dystrophy patients

**DOI:** 10.1186/s13023-021-01704-9

**Published:** 2021-01-30

**Authors:** Roman Panovský, Martin Pešl, Jan Máchal, Tomáš Holeček, Věra Feitová, Lenka Juříková, Lucia Masárová, Eva Pešlová, Lukáš Opatřil, Mary Luz Mojica-Pisciotti, Vladimír Kincl

**Affiliations:** 1 International Clinical Research Center, St. Anne’s Faculty Hospital, Brno, Czech Republic; 2 1St Department of Internal Medicine/Cardioangiology, St. Anne’s Faculty Hospital, Faculty of Medicine, Masaryk University Brno, Brno, Czech Republic; 3 Department of Biology, Faculty of Medicine, Masaryk University, Brno, Czech Republic; 4 Department of Pathophysiology, Faculty of Medicine, Masaryk University Brno, Brno, Czech Republic; 5 Department of Medical Imaging, St. Anne’s Faculty Hospital, Brno, Brno, Czech Republic; 6 Department of Pediatric Neurology, University Hospital Brno, Brno, Czech Republic; 7 First Department of Neurology, St. Anne’s Faculty Hospital, Faculty of Medicine, Masaryk University Brno, Brno, Czech Republic

**Keywords:** Cardiac magnetic resonance, Duchenne muscular dystrophy, Feature tracking, Strain analysis

## Abstract

**Background:**

Duchenne muscular dystrophy (DMD) manifests in males mainly by skeletal muscle impairment, but also by cardiac dysfunction. The assessment of the early phases of cardiac involvement using echocardiography is often very difficult to perform in these patients. The aim of the study was to use cardiac magnetic resonance (CMR) strain analysis and mitral annular plane systolic excursion (MAPSE) in the detection of early left ventricular (LV) dysfunction in DMD patients.

**Methods and results:**

In total, 51 male DMD patients and 18 matched controls were examined by CMR. MAPSE measurement and functional analysis using feature tracking (FT) were performed. Three groups of patients were evaluated: A/ patients with LGE and LV EF < 50% (n = 8), B/ patients with LGE and LVEF ≥ 50% (n = 13), and C/ patients without LGE and LVEF ≥ 50% (n = 30). MAPSE and global LV strains of the 3 DMD groups were compared to controls (n = 18).

Groups A and B had significantly reduced values of MAPSE, global longitudinal strain (GLS), global circumferential strain (GCS), and global radial strain (GRS) in comparison to controls (*p* < 0.05). The values of MAPSE (11.6 ± 1.9 v 13.7 ± 2.7 mm) and GCS (− 26.2 ± 4.2 v − 30.0 ± 5.1%) were significantly reduced in group C compared to the controls (*p* < 0.05).

**Conclusion:**

DMD patients had decreased LV systolic function measured by MAPSE and global LV strain even in the case of normal LV EF and the absence of LGE. FT and MAPSE measurement provide sensitive assessment of early cardiac involvement in DMD patients.

## Background

Duchenne and Becker muscular dystrophy (DMD and BMD) is an X-linked inherited muscular disease that can also severely affect the heart muscle with manifestation as a cardiomyopathy. Although the cardiac care of DMD patients has recently greatly improved, there are only limited data about detailed development of cardiomyopathy, with certain influence of specific mutation in the DMD gene [1, 2]. Despite multiple possible mechanisms of muscular dystrophy, regular heart examination and the preventive prescription of angiotensin-converting enzyme (ACE) inhibitors, as well as early initiation of the therapy have been shown to be effective [3]. However, detailed data on cardiomyopathy development as well as on optimal timing of therapy initiation are scarce [4, 5]. The treatment should likely be started no later than after the first signs of cardiac involvement are found [6–8].

Cardiovascular magnetic resonance (CMR) has become a main non-invasive diagnostic tool for DMD patients as the quality of echocardiographic gradually deteriorate during the disease progression, partially due to disease related skeletal deformities, but also narrow intercostal spaces [9, 10]. In addition to left ventricular (LV) global and regional function, late gadolinium enhancement (LGE) can image regional myocardial fibrosis. LGE usually appears in the earlier stages, even before a decrease of LV ejection fraction (EF) and predicts adverse cardiac events in DMD patients [11–13]. The assessment of LV systolic function is the most significant diagnostic and decision-making part of the examination. It is conventionally obtained by evaluation of the LV EF measurement and regional LV walls thickening. However, global LV EF and visible regional wall motion abnormalities may not be sensitive enough to detect incipient subtle changes in LV function. Recent studies have shown that incipient changes can be visualised as impaired LV longitudinal shortening assessed by mitral annular plane systolic excursion (MAPSE) [14–18] and, in particular, myocardial deformation measured using different approaches of strain analysis, could be affected earlier by slight incipient changes in LV function. Both these measures have been routinely assessed in different cohorts using echocardiography and the technical development in the CMR examination allows to assess them also using CMR. There are several CMR technique for the myocardial strain evaluation, including, tagging, feature tracking and tissue tracking. All of them were validated previously and used in different clinical scenarios, mainly to identify subclinical, early changes of LV function. In addition, these measures have a great advantage in the fact that could be acquired without the use of a gadolinium-based contrast agent [19–25]. Nevertheless, data on using strain analysis in patients with DMD are still limited [26–32] and MAPSE has not been studied in this population yet.

## Methods

The aim of the study was to use CMR strain analysis and MAPSE in the detection of early LV dysfunction in DMD patients.

### Patient population and cardiac MR data acquisition

The cohort of the Czech male population with genetically diagnosed DMD dystrophin mutation was screened in cooperation with the Muscular Dystrophy Registry REaDY (http://ready.registry.cz) [33] and enrolled according to the inclusion parameters as described previously [34]. All eligible patients fulfilling the inclusion criteria were included into the study. The inclusion criteria were: 1/ signed informed consent by the patient (or by the patient and his parents in the case of a child); 2/ absence of CMR contraindications such as an implanted pacemaker/defibrillator, cochlear implant, other ferromagnetic metal parts in the patient’s body, claustrophobia, etc.; 3/ absence of contraindications for using contrast media such as severe renal insufficiency; 4/ patient’s ability to co-operate during CMR examination; 5/ no known cardiovascular pathology apart from dystrophin cardiomyopathies. In total, 51 male DMD patients and 18 matched healthy controls were examined by CMR. Controls were males with a clinical indication for CMR by attending paediatricians with cardiology specialization, with normal CMR findings, normal other cardiac investigation results, and no other relevant medical history. The basic characteristics of the groups are shown in Table 1. The patient's inability to undergo CMR examination was the main reason for the patient's non-inclusion, particularly very small children (under 6 years old) on the one hand and older seriously ill males (advanced breathing problems, oxygen dependency, etc.) on the other.

**Table 1:** Basic characteristic of the study groups

	DMD n = 51	Controls n = 18	*P* value
Age (years)	14.7 ± 6.1	17.5 ± 2.9	0.064
Weight (kg)	49.1 ± 20.4	68.1 ± 10.5	**4.10**^**–4**^
Height (cm)	149 ± 19	177 ± 18	**4.10**^**–8**^
Dyspnoea (n (%))	7 (13.7%)	0 (0%)	0.18
BMI (kg/m^2^)	21.3 ± 5.6	21.8 ± 2.2	0.73
Hypertension (n (%))	3 (5.9%)	0 (0%)	0.56
Diabetes (n (%))	0 (0%)	1 (5.6%)	0.26
Corticosteroids (n (%))	22 (44.0%)	0 (0%)	**3.10**^**–4**^
ACE-inhibitors (n (%))	18 (36.0%)	0 (0%)	**2.10**^**–3**^
ARBs (n (%))	2 (4.0%)	0 (0%)	1.00
β-blockers (n (%))	8 (16.0%)	0 (0%)	0.10
Diuretics (n (%))	3 (6.0%)	0 (0%)	0.56

Continuous variables are expressed as the mean ± standard deviation, binary variables as count (percentage). The *p* values refer to the t-test in the case of continuous variables or to Fisher exact test in binary variables. *P*-values < 0.05 are marked in bold.

DMD, Duchenne muscular dystrophy; BMI, body mass index; ACE, angiotensin converting enzyme; ARB, angiotensin receptor blockers

CMR studies were performed according to the standard protocol using a 1.5 T scanner (Ingenia, Philips Medical Systems, Best, The Netherlands) and included functional imaging using balanced steady-state free precession (SSFP, b-TFE) cine sequences and LGE images acquired 10 min after an intravenous bolus of 0.2 mmol/kg of the gadolinium-based contrast agent gadobutrol (Gadovist, Bayer-Schering Pharma, Germany). CMR data acquisition was described in detail in the previous study [34].

For the detailed retrospective analysis, the patients were divided into groups based on LV EF and the presence or absence of LGE as an established marker. Four groups were compared – 1/ DMD patients with LGE and LV EF < 50% (group A), 2/ DMD patients with LGE and LV EF ≥ 50% (group B), 3/ DMD patients without LGE and preserved LV EF (group C), 4/ controls. MAPSE and global strain values were compared among the groups both unadjusted and after an adjustment for age. Further, the association of MR parameters with corticosteroid treatment was evaluated in DMD patients, to exclude possible confounding of the results.

To assess interobserver and intraobserver agreement, 13 random patients were blindly evaluated by two experienced observers (T.H and R.P.), one of them performed the analysis twice.

### MAPSE and strain analysis

Septal and lateral MAPSE was measured as previously described [10, 11] by defining end-diastolic and end-systolic mitral annular planes on a long-axis four-chamber view. The average MAPSE was calculated as the mean of septal and lateral MAPSE.

Myocardial strain analysis was performed using the feature tracking (FT) method by a commercially available software Image Arena (2D CPA MR, version 4.6.4.40, TomTec Imaging Systems GmbH, Unterschleissheim, Germany). Analyses were performed in a random and blinded order regarding the patient clinical characteristics. Both the endocardial and epicardial contours were manually traced in long-axis (2, 3 and 4-chamber views) and short-axis images (basal, midventricular and apical levels) during end-diastole (ED) and end-systole (ES). The basal level of the short-axis was taken as the first slice that showed the entire cardiac cycle without the appearance of the left ventricle outflow tract. The middle level of the short-axis was a slice located at the level of the papillary muscles, and the apical level was the last slice that showed the blood pool cavity throughout the entire cardiac cycle. The papillary muscles were excluded from the contours. The manually traced contours were propagated throughout the images for the complete cardiac cycle. The accuracy of this process was visually validated and corrected if necessary. In the case of significant suboptimal tracking, the analysis was repeated from the start to minimize variability [35].

The global LV strains were automatically measured and calculated. Short-axis images were used to determine the global circumferential strain (GCS) and global radial strain (GRS), while long-axis images (2, 3 and 4-chamber views) for the global longitudinal strain (GLS).

### Statistical analysis

For basic characteristics, the Fisher exact test was used to compare the binary parameters in DMD patients and controls, while the Student’s t-test for unmatched data was used to compare the continuous variables with a normal distribution. When comparing the CMR results and other data obtained in different groups of DMD patients to the control group, an ANOVA was used for continuous variables and χ^2^ test for categorical variables. In the case of a statistically significant result, the ANOVA was followed by Dunnet post hoc test and χ^2^ test by a series of 3 Fisher exact tests with Bonferroni correction. In the case of MAPSE and global strain values, the effect of age was also considered and Analysis of Covariance (ANCOVA) was employed with age as a cofactor. Normality was tested by the Kolmogorov–Smirnov test and visual inspection of the histograms.

To assess intraobserver and interobserver agreement in 10 randomly selected subjects, the intraclass correlation coefficient (ICC) was used. Pearson correlation coefficient was used for other correlations.

In all cases, results with a *p* value < 0.05 were considered statistically significant.

## Results

Table 1 shows a comparison of the selected basic characteristics of dystrophy patients and controls. DMD patients had lower body weight and height compared to controls and were treated by ACE-inhibitors and corticosteroids.

**Fig. 1:**
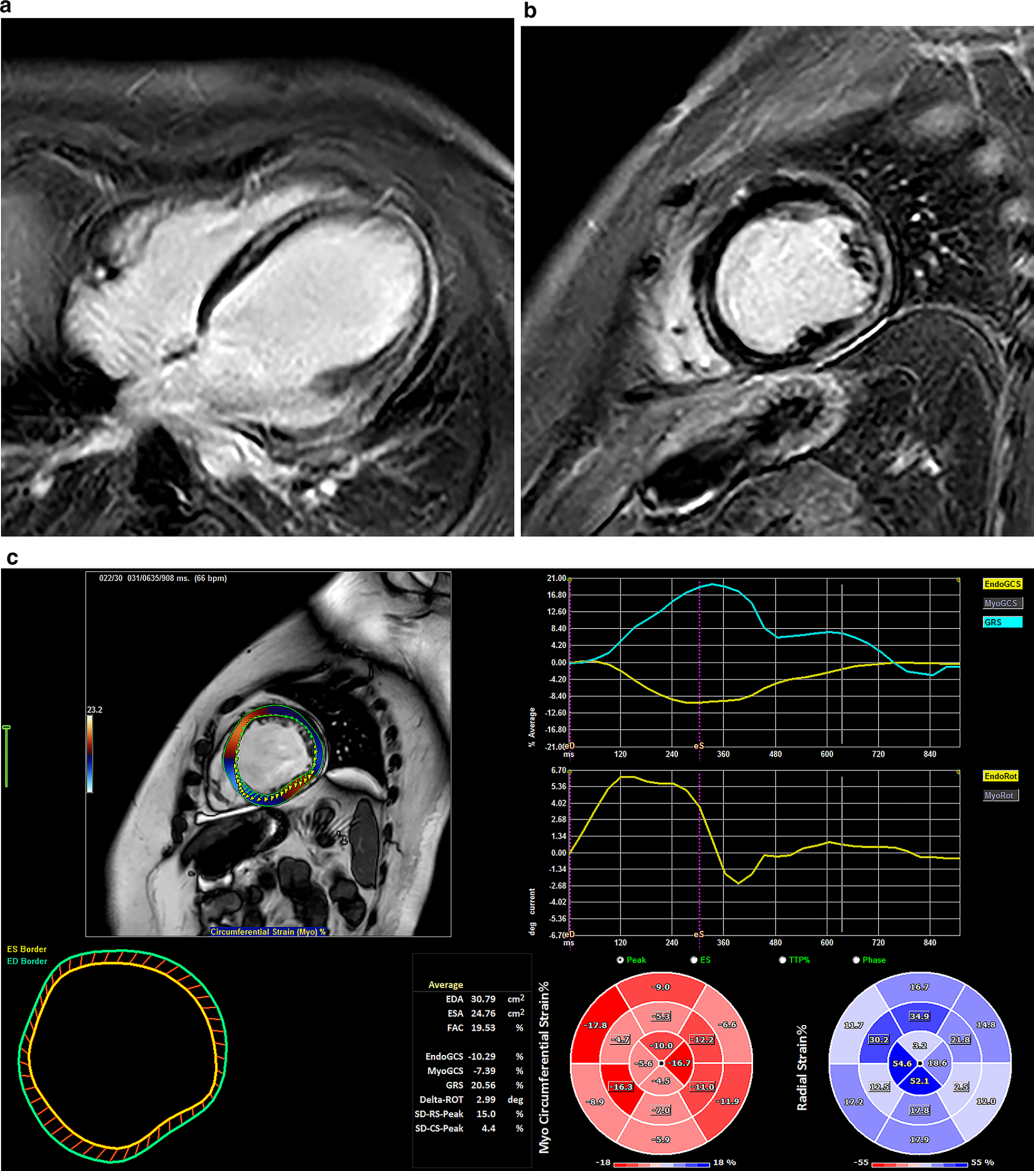
Cardiac involvement in Duchene Muscular Dystrophy patient. (**a**) late gadolinium enhancement of left ventricular myocardium in a 4-chamber view; (**b**) late gadolinium enhancement of left ventricular myocardium in a short-axis view; (**c)** Feature tracking analysis showing reduced global circumferential and global radial strain

Of the total 51 DMD patients, LV EF < 50% was found in 8 patients (16%), and LGE in 21 subjects (41%). Intramural and subepicardial LGE was found typically in the lateral wall (in all 21 subjects) with differing extension to other LV walls—to the inferior wall (9 patients) and/or to the interventricular septum and anterior wall (5 patients) (Fig. 1). No LGE was found in healthy controls. A comparison of the selected clinical and MR parameters among the groups is shown in Table 2. Not surprisingly, compared with other groups, patients in a group C were younger, and patients in groups B and C also had lower body weight. Similarly, corticosteroid treatment was more frequent in groups B and C, and cardiac treatment with ACE-inhibitors, β-blockers and diuretics was more frequent in advanced DMD patients. While patients in groups B and C had smaller LV (indexed LV end-diastolic volume 47.9 and 52.4 ml/m^2^), group A had larger LV (88.5 ml/m^2^) in comparison to the controls (64.8 ml/m^2^), and indexed LV stroke volume was lower in all 3 DMD groups.

**Table 2:** Comparison of selected parameters

	Group A (LGE + , EF < 50%) n = 8	Group B (LGE + , EF ≥ 50%) n = 13	Group C (LGE-, EF ≥ 50%) n = 30	Controls n = 18	*p*
Age (years)	20.6 ± 6.1	15.1 ± 4.8	13.0 ± 5.8*	17.5 ± 2.9	**8.10**^**–4**^
Weight (kg)	68.0 ± 19.9	52.6 ± 23.4*	42.1 ± 15.8*	68.1 ± 10.5	**6.10**^**–6**^
Height (cm)	166 ± 12	152 ± 19*	143 ± 18*	177 ± 18	**1.10**^**–9**^
BMI (kg/m^2^)	24.4 ± 5.9	22.4 ± 7.6	19.9 ± 4.1	21.8 ± 2.2	0.09
Dyspnoea (n (%))	3 (37.5%)*	3 (23.1%)	1 (3.5%)	0 (0%)	**7.10**^**–3**^
Hypertension (n (%))	3 (37.5%)*	0 (0%)	0 (0%)	0 (0%)	**3.10**^**–6**^
Diabetes (n (%))	0 (0%)	0 (0%)	0 (0%)	1 (5.6%)	0.42
Corticosteroids (n (%))	1 (12.5%)	6 (46.2%)*	15 (50.0%)*	0 (0%)	**8.10**^**–4**^
ACE-inhibitors (n (%))	5 (62.5%)*	8 (61.5%)*	5 (17.9%)	0 (0%)	**1.10**^**–4**^
ARBs (n (%))	2 (25.0%)	0 (0%)	0 (0%)	0 (0%)	**1.10**^**–3**^
β-blockers (n (%))	6 (75.0%)*	1 (7.7%)	1 (3.6%)	0 (0%)	**1.10**^**–7**^
Diuretics (n (%))	3 (37.5%)*	0 (0%)	0 (0%)	0 (0%)	**4.10**^**–6**^
LV EF (%)	37.9 ± 9.9*	61.2 ± 7.1	64.9 ± 6.8	66.3 ± 7.2	**1.10**^**–13**^
LVMi (g/m^2^)	59.2 ± 12.9	41.8 ± 7.7*	33.5 ± 7.6*	53.6 ± 13.0	**3.10**^**–11**^
LV EDVi (ml/m^2^)	88.5 ± 29.6*	47.9 ± 8.5*	52.4 ± 13.0*	64.8 ± 11.6	**8.10**^**–9**^
LV ESVi (ml/m^2^)	55.9 ± 23.9*	18.7 ± 5.12	19.1 ± 7.2	21.6 ± 4.4	**7.10**^**–14**^
LV SVi (ml/m^2^)	32.6 ± 9.5*	29.1 ± 5.4*	32.8 ± 8.2*	43.3 ± 10.4	**1.10**^**–5**^

Continuous variables are expressed as the mean ± standard deviation; categorical as total count (percentage)

Variables marked in bold have their ANOVA/χ^2^ test *p* value < 0.05

^*^denotes a significant difference compared with the control group (Dunnet post hoc test or a series of 3 Fisher exact tests with Bonferroni correction)

BMI, body mass index (kg/m^2^); ACE, angiotensin converting enzyme; ARB, angiotensin receptor blockers; LGE + , positive late gadolinium enhancement; LGE–, negative late gadolinium enhancement; LV, left ventricle; EF, ejection fraction; LVMi, left ventricular mass index; EDVi, end-diastolic volume index; ESVi, end-systolic volume index; SVi, stroke volume index

Groups A and B had significantly reduced values of MAPSE, GCS, and GRS in comparison to the controls (*p* < 0.05). In addition, values of MAPSE (11.6 ± 1.9 v 13.7 ± 2.7 mm), and GCS (− 26.2 ± 4.2 v − 30.0 ± 5.1%) were significantly reduced even in group C compared to the control group (*p* < 0.05), although both groups had similar LV EF (65.9 ± 6.8 and 66.3 ± 7.2, *p* = NS). The comparison of global strain values and MAPSE is shown in Table 3. The differences in global strain values and MAPSE were the same after adjustment for patient’s age, which seemed to play negligible role in observed differences (Table 4). The values of MAPSE did not correlate with age in either DMD patients (r = -0.02; *p* = 0.91) or controls, where a weak, but nonsignificant negative trend was found (r = -0.20; *p* = 0.47). Similarly, no significant correlation with age was found for global strain values in DMD patients and controls (r < 0.25; *p* > 0.10 in all cases), with the exception of GCS in DMD patients (r = 0.41; *p* = 0.004).

**Table 3:** Comparison of global left ventricular strains and MAPSE (crude)

	Group A (LGE + , EF < 50%) n = 8	Group B (LGE + , EF ≥ 50%) n = 13	Group C (LGE−, EF ≥ 50%) n = 30	Controls n = 18	*p* (A vs. controls)	*p* (B vs controls)	*p* (C vs. controls)
GLS (%)	− 14.94 ± 1.91	− 19.14 ± 2.57	− 21.9 ± 4.12	− 20.91 ± 3.05	**3.10**^**–4**^	0.39	0.70
GCS (%)	− 14.71 ± 4.18	− 24.81 ± 5.74	− 26.17 ± 4.19	− 30.03 ± 5.12	**1.10**^**–8**^	**0.013**	**0.033**
GRS (%)	38.3 ± 9.80	50.45 ± 14.22	60.02 ± 15.21	67.41 ± 10.67	**1.10**^**–5**^	**3.10**^**–3**^	0.21
MAPSE (mm)	10.50 ± 1.10	10.77 ± 1.55	11.63 ± 1.88	13.66 ± 2.70	**2.10**^**–3**^	**7.10**^**–4**^	**5.10**^**–3**^
MAPSE lateral (mm)	11.00 ± 1.31	11.31 ± 2.18	12.60 ± 2.24	14.44 ± 3.27	**5.10**^**–3**^	**3.10**^**–3**^	0.05
MAPSE septal (mm)	10.00 ± 1.07	10.23 ± 1.59	10.67 ± 1.83	12.88 ± 2.33	**2.10**^**–3**^	**9.10**^**–4**^	**9.10**^**–4**^

Variables are expressed as the mean ± standard deviation

Variables marked in bold have their ANOVA p value < 0.05

*P* values of the Dunnet post hoc test < 0.05 are marked in bold

LGE +, positive late gadolinium enhancement; LGE–, negative late gadolinium enhancement; LV, left ventricle; EF, ejection fraction; GLS, global longitudinal strain; GCS, global circumferential strain; GRS, global radial strain; MAPSE, mitral annular plane systolic excursion

**Table 4:** Comparison of global left ventricular strains and MAPSE (age-adjusted)

	Group A (LGE + , EF < 50%) n = 8	Group B (LGE + , EF ≥ 50%) n = 13	Group C (LGE−, EF ≥ 50%) n = 30	Controls n = 18	*p* (A vs. controls)	*p* (B vs controls)	*p* (C vs. controls)
GLS (%)	− 14.94 ± 1.91	− 19.14 ± 2.57	− 21.9 ± 4.12	− 20.91 ± 3.05	**3.10**^**–4**^	0.38	0.70
GCS (%)	− 14.71 ± 4.18	− 24.81 ± 5.74	− 26.17 ± 4.19	− 30.03 ± 5.12	**1.10**^**–8**^	**0.013**	**0.034**
GRS (%)	38.3 ± 9.80	50.45 ± 14.22	60.02 ± 15.21	67.41 ± 10.67	**1.10**^**–5**^	**3.10**^**–3**^	0.21
MAPSE (mm)	10.50 ± 1.10	10.77 ± 1.55	11.63 ± 1.88	13.66 ± 2.70	**2.10**^**–3**^	**8.10**^**–4**^	**5.10**^**–3**^
MAPSE lateral (mm)	11.00 ± 1.31	11.31 ± 2.18	12.60 ± 2.24	14.44 ± 3.27	**6.10**^**–3**^	**3.10**^**–4**^	0.05
MAPSE septal (mm)	10.00 ± 1.07	10.23 ± 1.59	10.67 ± 1.83	12.88 ± 2.33	**2.10**^**–3**^	**8.10**^**–4**^	**8.10**^**–4**^

Variables are expressed as the mean ± standard deviation

Variables marked in bold have their ANCOVA *p* value < 0.05

*P* values of the Dunnet post hoc test < 0.05 are marked in bold

LGE +, positive late gadolinium enhancement; LGE–, negative late gadolinium enhancement; LV, left ventricle; EF, ejection fraction; GLS, global longitudinal strain; GCS, global circumferential strain; GRS, global radial strain; MAPSE, mitral annular plane systolic excursion

To evaluate the possible association of corticosteroid treatment, which could stand as a confounder, with MAPSE and global strain values, the parameters were compared between the corticosteroid-treated and untreated patients. All patients except of one belonged to group B or C. The MAPSE and global strain values of patients from groups B and C who were treated or not by corticosteroids are shown in Table 5. There was no difference either in DMD patients as a whole or in any of the subgroups A, B or C (*p* > 0.10 in all cases), with the exception of septal MAPSE in group C, where a trend towards lower value in corticosteroid-treated patients (10.07 ± 1.53 vs. 11.23 ± 1.54; *p* = 0.056), insignificant at α = 0.05, was observed.

**Table 5:** Comparison of MAPSE and global strain values in patients treated and not treated by corticosteroids

	Group B (LGE + , LV EF ≥ 50%) n = 13				Group C (LGE −, LV EF ≥ 50%) n = 30		
		Corticosteroid-treated (n = 6)	No corticosteroids (n = 7)	*p* value	Corticosteroid-treated (n = 15)	No corticosteroids (n = 15)	*p* value
GLS (%)		− 19.15 ± 1.86	− 19.14 ± 3.22	1.00	− 20.95 ± 3.63	− 23.32 ± 4.61	0.16
GCS (%)		− 26.51 ± 5.38	− 23.36 ± 6.04	0.35	− 25.19 ± 3.53	− 27.74 ± 4.73	0.14
GRS (%)		55.34 ± 10.69	46.26 ± 16.28	0.27	56.70 ± 16.75	63.80 ± 14.53	0.27
MAPSE (mm)		10.83 ± 1.13	10.71 ± 1.93	0.90	11.23 ± 1.66	12.08 ± 1.90	0.22
MAPSE lateral (mm)		11.00 ± 1.90	11.57 ± 2.51	0.66	12.40 ± 1.99	12.92 ± 2.56	0.55
MAPSE septal (mm)		10.67 ± 1.21	9.86 ± 1.86	0.38	10.07 ± 1.53	11.23 ± 1.54	0.06

Variables are expressed as the mean ± standard deviation

LGE +, positive late gadolinium enhancement; LGE–, negative late gadolinium enhancement; LV, left ventricle; EF, ejection fraction; GLS, global longitudinal strain; GCS, global circumferential strain; GRS, global radial strain; MAPSE, mitral annular plane systolic excursion

Inter- and intra-observer variability analysis was performed retrospectively on 13 subjects. In almost all parameters, the intraobserver ICC was > 80% and interobserver ICC was > 70%, with the notable exception of GRS, where the agreement was poor (ICC < 10%). Therefore, the results of GRS must be interpreted with caution. The values for lateral and septal MAPSE, as well as for GCS, GLS and GRS are shown in Table 6.

**Table 6:** Intra-rater and inter-rater agreement in selected parameters

	ICC (intra-rater)	ICC (inter-rater)
GLS (%)	0.80	0.82
GCS (%)	0.90	0.77
GRS (%)	0.03	0.03
MAPSE lateral (mm)	0.96	0.93
MAPSE septal (mm)	0.97	0.95

ICC, intraclass correlation coefficient; GLS, global longitudinal strain; GCS, global circumferential strain; GRS, global radial strain; MAPSE, mitral annular plane systolic excursion

## Discussion

To the best of our knowledge, this is the first study assessing CMR-derived MAPSE in the DMD population and so far one of the largest studies using CMR-derived FT in DMD males. The study highlights some remarkable findings. Firstly, it confirmed a premise that the myocardium of DMD patients presents with early, subclinical cardiomyopathy long before detectable LV EF decline and/or LGE. GCS and MAPSE were significantly reduced in DMD patients even at the stage of normal LV EF and absence of LGE. Secondly, it showed the utility of CMR FT strain analysis and MAPSE measurement in these patients and their potential as new imaging biomarkers of early myocardial involvement in DMD subjects.

Regarding FT-derived myocardial strain in DMD subjects, there are only a few published studies for comparison. Siegel et al. [27] demonstrated similar results regarding the reduction of GCS. Twenty-four DMD patients were examined by FT and echo-based speckle tracking (STE) strain measurement. The average circumferential strain measured by FT was significantly reduced compared to controls (− 18.8 vs − 25.5—slightly lower values compared to ours) and moreover differed between LGE positive and negative subjects. CMR FT was able to find a difference between DMD and controls even in patients where STE did not detect significant deformation decline. Circumferential myocardial strain assessed using FT in DMD patients was also studied by Hor et al. [26]. The FT-based assessment was compared to the tagging-based harmonic phase (HARP), and a high correlation was found between the two techniques. In line with our results, DMD patients with normal LV EF and an absence of LGE had decreased GCS.

A similar CMR technique for myocardial strain analysis, tissue tracking was used in a study by Buddhe [31] for comparison of echocardiographic and CMR parameters in 33 DMD subjects. They found a weak correlation between echocardiographic parameters and CMR parameters including strain analysis and concluded that CMR should be performed routinely in children with DMD, not only for LGE imaging but also for functional assessment. They also described decreased GLS, GCS, and GRS values in patients with lower LV EF, however, no controls were compared to DMD patients with normal LV EF.

In agreement with previous studies, circumferential strain seems to be the most sensitive strain marker for early myocardial changes in DMD. In comparison to the longitudinal and radial ones, the circumferential strain was also significantly reduced in LGE negative patients with normal LV EF. The reason for this fact is not fully understood. Nevertheless, it was previously speculated that a gradual loss of sarcomeres could lead to changes in contractility, at first manifesting by the deterioration of circumferential function [31, 32]. In contrast, STE could detect similar subclinical stages of cardiomyopathy, but longitudinal strain decrease was more evident as a first marker of dilated cardiomyopathy [36] or heart transplant rejection [37].

Several antecedent studies have used CMR tagging for strain assessment in the DMD population [28–30, 32, 38]. The studies were limited to only circumferential strain analysis and this generally showed its utility in the diagnosis of early cardiomyopathy. Nevertheless, tagging techniques require an additional scanning sequence which could mean a prolonged scanning time, and also the impossibility to perform retrospective analysis in patients where these images were not acquired. On the other hand, FT can be performed on routinely acquired SSFP images, and so it can be easier to use.

Until now, no study using CMR-derived MAPSE assessment in DMD patients has been published. However, LV long axis dysfunction is known as an early biomarker of some pathological states as well as an independent prognostic predictor incremental to common risk factors including LV EF [16–18]. MAPSE could also be assessed using routine cardiac cine imaging without the need for additional scanning or special software and is easily measured with low interobserver variability.

The study supported the evidence that the relatively simple CMR parameters, FT-derived strain and MAPSE could recognize early myocardial changes in DMD patients even without the use of a contrast agent or at least limit the frequency of gadolinium-based contrast agent exposure [39]. Besides lower use of contrast agent, this approach using MAPSE and myocardial strain could also lead to shorter scan time and less medical costs. Above that, CMR overcomes typical imaging limitations of echocardiography in DMD patients. These parameters could also be potentially used as sensitive biomarkers for early initiation and follow-up monitoring of the cardiovascular therapy including the assessment of the therapeutic effect [40].

The study has several limitations. This is a single-center, retrospective study, with a limited number of patients because DMD is a rare disease. Actually, almost the whole cohort of the Czech male population with genetically diagnosed DMD dystrophin mutation (more than 100 males) was screened, and all eligible patients fulfilling inclusion criteria were examined using CMR. Another limitation is the fact that only global myocardial strains were evaluated. Nevertheless, it is known that the FT technique has limited reproducibility of segmental strain measurements, thus FT regional analysis is not recommended for clinical and research purposes [25], although segmental values would be certainly very interesting. Still, the alternative assessment of regional function by echocardiography is usually also quite challenging due to limited image quality.

## Conclusion

DMD patients had decreased LV longitudinal systolic function measured by MAPSE and GCS even in the case of normal LV EF and absence of LGE. CMR-derived FT and MAPSE measurement provide a sensitive assessment of early cardiac involvement in DMD patients.

## Data Availability

The datasets analyzed during the current study are available from the corresponding author upon reasonable request.

## Abbreviations

ACE: Angiotensin converting enzyme
ANOVA: Analysis of variance
ARB: Angiotensin receptor blockers
BMD: Becker muscular dystrophy
BMI: Body mass index
b-TFE: Balanced turbo-field echo
CMR: Cardiac magnetic resonance
DMD: Duchenne muscular dystrophy
ED: End-diastole
EDVi: End-diastolic volume index
EF: Ejection fraction
ES: End-systole
ESVi: End-systolic volume index
FT: Feature tracking
LGE: Late gadolinium enhancement
LGE +: Positive late gadolinium enhancement
LGE −: Negative late gadolinium enhancement
LV: Left ventricle
LVMi: Left ventricular mass index
MAPSE: Mitral annular plane systolic excursion
mm: Millimetre
SSFP: Steady state free precession
SVi: Indexed stroke volume
